# Genome-Wide Analysis of Protein-Protein Interactions and Involvement of Viral Proteins in SARS-CoV Replication

**DOI:** 10.1371/journal.pone.0003299

**Published:** 2008-10-01

**Authors:** Ji'An Pan, Xiaoxue Peng, Yajing Gao, Zhilin Li, Xiaolu Lu, Yingzhao Chen, Musarat Ishaq, Dan Liu, Marta L. DeDiego, Luis Enjuanes, Deyin Guo

**Affiliations:** 1 State Key Laboratory of Virology and Modern Virology Research Centre, College of Life Sciences, Wuhan University, Wuhan, People's Republic of China; 2 Centro Nacional de Biotecnología, CSIC, Department of Molecular and Cell Biology, Campus Universidad Autónoma, Madrid, Spain; University of Wyoming, United States of America

## Abstract

Analyses of viral protein-protein interactions are an important step to understand viral protein functions and their underlying molecular mechanisms. In this study, we adopted a mammalian two-hybrid system to screen the genome-wide intraviral protein-protein interactions of SARS coronavirus (SARS-CoV) and therefrom revealed a number of novel interactions which could be partly confirmed by in vitro biochemical assays. Three pairs of the interactions identified were detected in both directions: non-structural protein (nsp) 10 and nsp14, nsp10 and nsp16, and nsp7 and nsp8. The interactions between the multifunctional nsp10 and nsp14 or nsp16, which are the unique proteins found in the members of *Nidovirales* with large RNA genomes including coronaviruses and toroviruses, may have important implication for the mechanisms of replication/transcription complex assembly and functions of these viruses. Using a SARS-CoV replicon expressing a luciferase reporter under the control of a transcription regulating sequence, it has been shown that several viral proteins (N, X and SUD domains of nsp3, and nsp12) provided in *trans* stimulated the replicon reporter activity, indicating that these proteins may regulate coronavirus replication and transcription. Collectively, our findings provide a basis and platform for further characterization of the functions and mechanisms of coronavirus proteins.

## Introduction

Interactions between viral proteins play pivotal roles in many processes during the viral infection cycle. This is the case in the formation of virus replication complexes, coordinated functions between different viral proteins, assembly of virions, and counter-defense of host immune responses. Analysis of protein-protein interactions is essential to understand protein functions and the molecular mechanisms underlying biological processes. As the viral genomes are of limited sizes, they are particularly well suited for genome-wide analysis of all possible protein-protein interactions. However, the viral protein interaction maps have been generated until now only for a limited number of viruses, including T7 bacteriophage [1], vaccinia virus [2], potato virus A [3], pea seed-borne mosaic virus [3], wheat steak mosaic virus [4], hepatitis C virus [5], [6], porcine teschovirus [7], Kaposi sarcoma-associated herpesvirus [8], and very recently severe acute respiratory syndrome coronavirus (SARS-CoV) [9], [10].

Although a large variety of methods have been developed to detect protein-protein interactions, only a few of them are suited for large-scale and high throughput protein interaction analysis. Until now, all the genome-wide analysis of protein interaction networks for viruses and cells have been carried out mainly with the yeast two-hybrid systems, in combination with glutathione-S-transferase (GST) pull-down assays to verify major interactions [8], [9]. Considering that protein modifications that are likely to influence protein interactions may be different for certain proteins in the context of yeast and mammalian cells, the mammalian two-hybrid system may better reflect genuine protein interactions for human viruses. Accordingly, we adopted the mammalian two-hybrid system for detecting genome-wide protein-protein interactions of SARS-CoV.

The coronaviruses are classified into the family *Coronaviridae* in the order *Nidovirales* and possess the largest RNA genomes known. The genome of SARS-CoV contains a single-stranded, plus-sense RNA of approximately 29.7 kb in length. Fourteen open reading frames (ORFs) have been identified, of which 12 are located in the 3′ end of the genome [11], [12]. The two large ORFs (1a and 1b) in the 5′-proximal two-third of the genome encode the viral replicase and are translated directly from the genomic RNA, while ORF 1b is expressed by −1 ribosomal frameshifting. The large polypeptides encoded by 1a and 1b are considered to be cleaved into 16 functional replicase proteins by two proteinases, a papain-like proteinase 2 encoded by nsp3 and a 3C-like proteinase (or main proteinase) encoded by nsp5 [12].

A number of functions or characteristics have been identified for the 16 non-structural proteins (nsps). Nsp1 was proved to be able to suppress host gene expression by promoting host mRNA degradation and was involved in cellular chemokine deregulation [13], [14]. Nsp2 seems not to play a crucial role in the generation of infectious viruses in cell culture [15]. Nsp3 is involved in many activities including papain-like proteinase activity, deubiquitinating activity, and ADP-ribose-1″-phosphatase activity [16]–[18], [60], which are essential for viral replication and transcription. Nsp5 encodes a 3C-like proteinase which is considered to be an important target for antiviral drug design [19]. Coronavirus nsp4 and nsp6 are transmembrane proteins that could anchor the replication complexes to double membrane vesicles [20]. Based on the structural analysis, hexadecamer of nsp7 and nsp8 may possess dsRNA-binding activity [21]. Nsp8 was shown to have RNA-dependent RNA polymerase (RdRp) activity that could be involved in producing primers utilized by nsp12 which is normally accepted to be the RdRp for SARS-CoV [22], [23]. Nsp9 is a single-stranded RNA-binding protein [24]. Though the structure of nsp10 is resolved, its function is still poorly understood, except that of nsp10 of MHV, homologous to that of SARS-CoV, which regulates viral RNA synthesis [25]–[27]. Nsp13 is shown to be RNA helicase and 5′-triphosphatase that may play a crucial role in the viral RNA capping [28], [29]. Nsp14 of coronaviruses possesses a 3′->5′ exoribonuclease activity which may be involved in the proof-reading ability during the viral RNA replication and transcription [30]–[32]. Besides the exoribonuclease activity, SARS-CoV also possesses the endoribonuclease activity that is rendered by nsp15 [33]. According to the bioinformatic prediction, nsp16 and SUD domain of nsp3 of SARS-CoV may function as 2′-O methyltransferase and guanine N7 methyltransferase, respectively [34], [35]. Very recently, the 2′-O methyltransferase activity was confirmed experimentally for nsp16 of feline coronavirus [36]. Nsp14, 15 and 16 were shown to be essential for efficient replication of coronavirus [30], [37], [38].

The 3′ one-third of genome encodes 12 viral proteins including the structural and accessory proteins, which are translated from about 10 subgenomic RNAs [39], [40]. Proteins S, E, M, and N are four well described structural proteins, and 3a, 6, 7a, and 7b were also reported to be virion-associated proteins or viral structural proteins [41]–[44]. Multiple functions and activities have been identified for the structural and accessory proteins, including apoptosis induction, interference with the innate immunity response, and regulating the cellular protein expression [45]. However, these proteins are not essential for viral replication and transcription at least in cell culture and tested animal models, except nucleocapsid (N) protein [46]–[49].

Previous studies on SARS-CoV focused mostly on the molecular characterization and functional analysis of individual proteins encoded by SARS-CoV and limited information is available on viral protein-protein networks of coronaviruses [50]. To provide more insights into the functions of individual proteins, we analyzed interactions between all SARS-CoV-encoded proteins. By using mammalian two-hybrid assays, 40 different interactions between viral proteins have been identified, and six novel interactions could be confirmed in vitro by biochemical assays. Moreover, a sensitive replication and transcription reporter system of SARS-CoV was established in this study, and, based on this system, we examined the impacts of all the individual viral proteins on the viral replication and transcription and found that the N protein played an important role at the early stage of SARS-CoV genome replication.

## Results

### Identification of protein-protein interactions of SARS-CoV based on mammalian two-hybrid assays

In this study, a mammalian two-hybrid system was adopted to analyze the protein-protein interactions of SARS-CoV as it was assumed that the viral proteins expressed in mammalian cells were prone to be in their native conformations and therefore the interactions detected were more likely to be biologically relevant in comparison with other in vitro biochemical methods and assays performed in yeast cells [51].

For analysis of genome-wide protein interactions of SARS-CoV, all known ORFs were amplified by PCR from viral cDNAs of SARS-CoV isolate WHU [39] and cloned into pGEM-T vector (Table 1). The large polyprotein encoded by ORF 1a/b was split into 18 domains according to the proteinase cleavage sites, except for nsp3 which was divided into 3 parts based on predicted functional domains. For structural and accessory proteins, the intact ORFs including start and stop codons were amplified, except for S protein which was divided into S1 and S2 as these domains were proved to be two separate domains with distinct functions [52]. All the primers used for amplification were designed by inserting appropriate restriction sites which could be used for subcloning all the fragments from pGEM-T cloning vectors into mammalian two-hybrid vectors pM (bait) and pVP16 (prey) and other protein expression vectors (see below) in correct reading frames.

**Table 1:** The sequences of SARS-CoV used for interaction analysis.

*Protein*	*Coding sequence* *	*Position in polyprotein* *	*Protein length*	*Function described* **
nsp1	265–804	M1-180G	180	Regulation of host gene expression
nsp2	805–2718	A181-G818	638	
nsp3.1	2719–4896	A819-T1544	726	ADRP, SUD for OGB
nsp3.2	4897–7035	I1545-D2257	713	PLpro, DU
nsp3.3	7036–8484	F2258-G2740	483	
nsp4	8485–9984	K2741- Q3240	500	TM
nsp5	9985–10902	S3241- Q3546	306	3CLpro
nsp6	10903–11772	G3547- Q3836	290	TM
nsp7	11773–12021	S3837-Q3919	83	dsRNA-binding
nsp8	12022–12615	A3920-Q4117	198	dsRNA-binding & RdRp
nsp9	12616–12954	N4118-Q4230	113	ssRNA-binding
nsp10	12955–13371	A4231-Q4369	139	Regulation of viral RNA synthesis
nsp10–11	12955–13410	A4231-Q4382	152	
nsp11	13372–13410	S4370-V4382	13	
nsp12	13372–16166	S4370-Q5301	932	RdRp
nsp13	16167–17969	A5302-Q5902	601	Hel, NTPase
nsp14	17970–19550	A5903-Q6429	527	ExoN
nsp15	19551–20588	S6430-Q6775	346	XendoU
nsp16	20589–21482	A6776-N7073	298	2′-O-MT
S1	21492–23531	n/a	680	spike (receptor-binding)
S2	23532–25259	n/a	575	spike (fusion peptide,& TM)
3a	25268–26092	n/a	274	TM, ion channel
3b	25689–26153	n/a	154	TM, antagonist of IFN
E	26117–26347	n/a	76	envelope
M	26398–27063	n/a	221	membrane
6	27074–27265	n/a	63	antagonist of IFN
7a	27273–27641	n/a	122	
7b	27638–27772	n/a	44	
8b	27864–28118	n/a	39	
N	28120–29388	n/a	422	Nucleocapsid, antagonist of IFN
9b	28130–28426	n/a	98	

* The coordinate of the sequence is based on the genome of SARS-CoV WHU (GenBank accession number: AY394850). n/a: not applied.

** Abbreviations: ADRP, adenosine diphosphate-ribose 1″-phosphatase; SUD, SARS Unique Domain; OGB, oligo(G)-binding; PLpro, papain-like cysteine proteinase; DU, deubiquitinating activity; TM, transmembrane domain; 3CLpro, 3C-like cysteine proteinase; RdRp, RNA-dependent RNA polymerase; Hel, 5′ to 3′ RNA helicase; NTPase, NTP and RNA 5′ triphosphatase; ExoN, 3′ to 5′ exonuclease; XendoU, endoribonuclease; 2′-O-MT, S-adenosylmethionine-dependent ribose 2′-O-methyltransferase; IFN, interferon.

**Figure 1:**
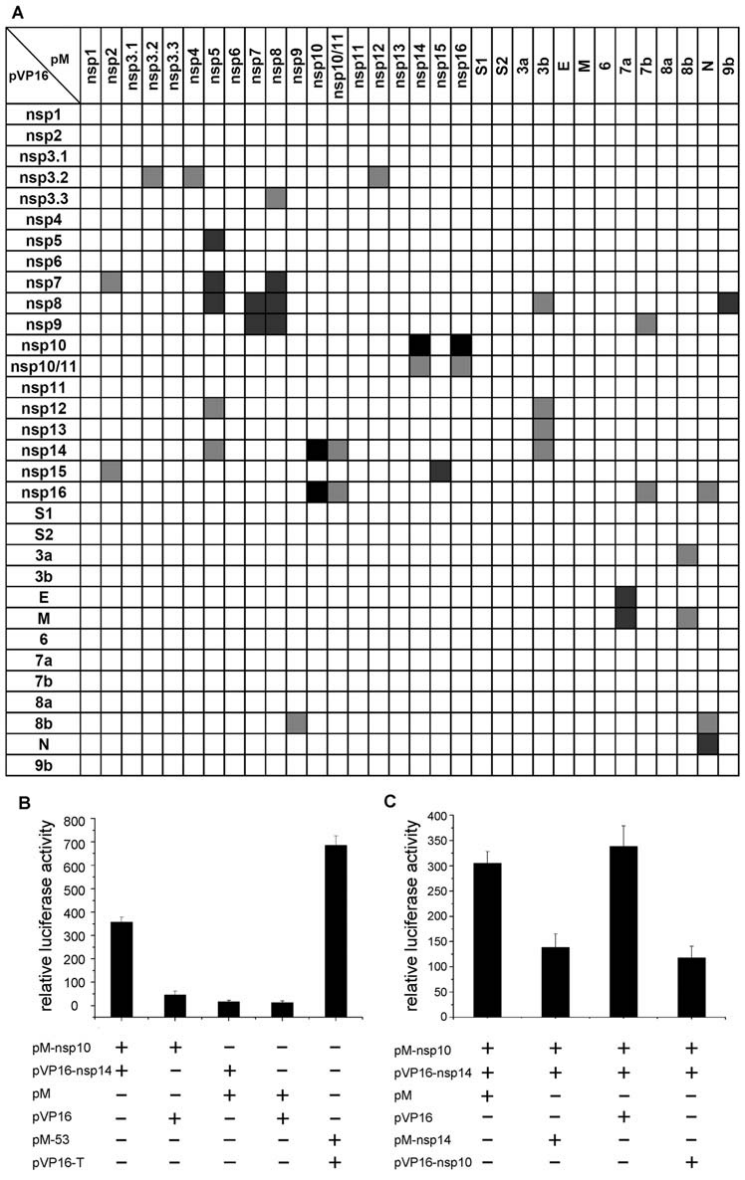
Protein interactions of SARS-CoV detected by mammalian two-hybrid assays. (A) Interaction matrix of SARS-CoV proteins. The grey squares indicate the novel interactions detected in this work. The black squares represent the interactions which have also been reported previously, including nsp5–nsp5[19], [68], [69], nsp5–nsp7 and nsp8[9], nsp7–nsp7[21], [70], nsp7–nsp8[21], nsp7–nsp9[9], nsp8–9b[9], nsp10–nsp14 and nsp10–nsp16[10], nsp15–nsp15 [54], [56], [71], [72], nsp7-E and 7a-M[73], N-N[55], [57] and N-N[55], [57]. (B) A typical result for a positive interaction with the example of nsp10–nsp14. The combination of pM-53 and pVP16-T represents a positive control. (C) A typical interaction inhibition assay performed to confirm that the interaction was not resulted from self-activation. Error bars represent standard deviations from three independent experiments.

In total, 1024 interaction combinations between all SARS-CoV proteins were examined in a pairwise matrix. As a result, 40 different interactions were detected using the mammalian two-hybrid assays (Fig. 1A). All the interactions shown (Fig. 1) were most likely specific for the viral protein domain of the fusion proteins as the activities of the reporter genes were reduced to background levels when one interacting partner in any of the combinations was replaced with non-relevant fusion protein (negative control) (data not shown). To further show the specificity of the interactions (Fig. 1), a quantitative assay for a typical positive interaction exemplified by nsp10–nsp14 and various controls were shown (Fig. 1B). To test the specificity of individual interactions, competitive assays were performed by co-expression of viral proteins using a different vector within the cells transformed with the bait and prey constructs. The co-expressed partner proteins interfered with the nsp10–nsp14 interaction, leading to reduced reporter activity (Fig. 1C), indicating that the same proteins with different fusion domains competed with each other and impaired the specific interactions needed for activation of the reporter genes.

Most interactions showed directionality, indicating the influence of fusion domains on the interacting sites. Nevertheless, three pairs of interactions were detected in both directions: nsp10 and nsp14, nsp10 and nsp16, and nsp7 and nsp8. Nsp11 is a small polypeptide containing only 13 amino acids and no interaction was detected with it in various assays but nsp11 in the fusion with nsp10 (nsp10/11) could significantly enhance the binding capability of nsp10 with either nsp14 or nsp16, indicating that the small nsp11 may also play important roles in viral protein interactions and replication. Self-interactions of nsp3, nsp5, nsp8, nsp15 and N were observed, suggesting that these proteins could form dimeric or multimeric complexes by interacting with themselves. Furthermore, 8 interactions between non-structural proteins and accessory proteins (3b, 7b, 8b, 9b) and one interaction between non-structural protein nsp16 and structural protein N were detected, indicating that some of the accessory proteins might be involved in viral replication and transcription processes. Nevertheless, that being the case, these interactions could have a minor effect on modulating virus replication as the virus-specific proteins are not strictly essential for viral replication at least in cell culture and mouse models [48], [49].

### Confirmation of SARS-CoV protein interactions by pull-down assays

**Figure 2:**
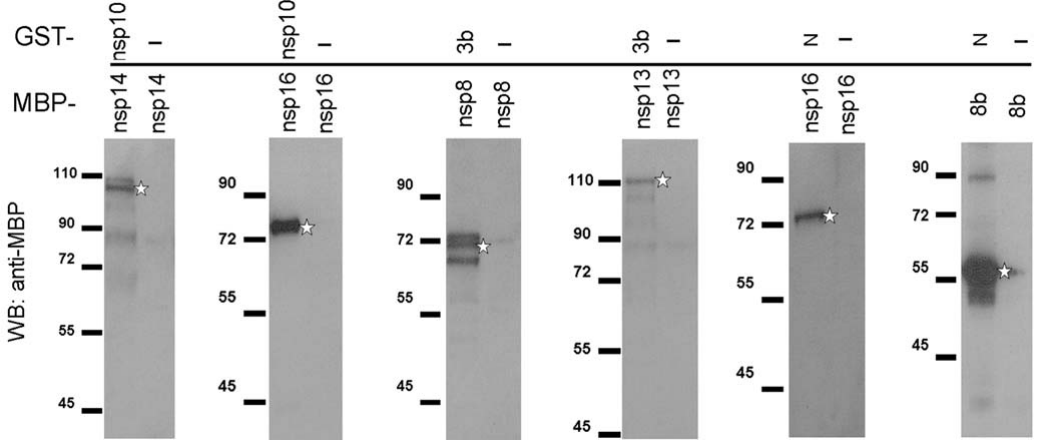
Confirmation of the novel interactions by pull-down assays. The two test proteins were fused with glutathione S-transferase (GST) and maltose-binding protein (MBP), respectively, and mixed for binding in PBS buffer as described in the Materials and Methods section. The protein mixture was pulled down with glutathione-Sepharose that binds GST and GST fusion proteins. Proteins bound by glutathione-Sepharose were resolved in SDS-PAGE, transferred to PVDF membrane and then was detected by anti-MBP rabbit serum. For every assay, GST protein was used as a negative control. For example, to examine the interaction between nsp10 and nsp14, the mixtures of GST-nsp10/MBP-nsp14 and GST/MBP-nsp14 were incubated with glutathione-Sepharose and the proteins pulled down by glutathione-Sepharose were identified by anti-MBP rabbit serum, respectively. The proteins indicated on the left side of the vertical line were MBP fusions and that on the right are GST fusions with “-” indicating non-fused GST as negative control. The star signs indicate the expected bands for MBP-fusion proteins. The smaller bands observed are probably premature proteins or degradation products of the same proteins.

To confirm the interactions which were newly detected by mammalian two-hybrid assays, pull-down assays were performed. Proteins that were related to the interactions not reported previously were expressed in different bacterial systems including pET30a (His-tagged), pGEX-6P-1 (GST fusion) and pMAL-c2X (MBP fusion). Using pull-down assays that were described in Materials and Methods section, six protein-protein interactions were confirmed, including those between: nsp10 and nsp14, nsp10 and nsp16, nsp13 and 3b, nsp8 and 3b, nsp16 and N, and 8b and N (Fig. 2). Due to difficulties in bacteria expression, the interactions involving nsp12, nsp3.2 and nsp3.3 could not be confirmed.

### Establishment of a reporter gene-containing replicon and analysis of the impacts of viral proteins provided in *trans* on viral replication/transcription of SARS-CoV

**Figure 3:**
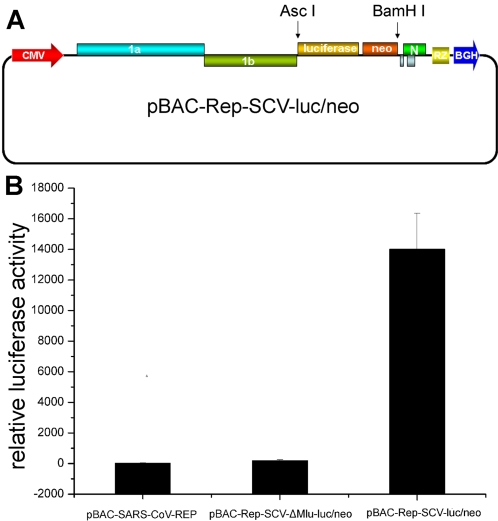
Structure and activity assay of the reporter replicon construct pBAC-Rep-SCV-luc/neo. (A) Schematic structure of the replicon. The coding sequence of luciferase-neomycin fusion under the control of M gene TRS was inserted into the basic replicon construct pBAC-SARS-CoV-REP between AscI and BamHI sites (For details, see the Materials and Methods). (B) Luciferase assays of the reporter replicons. 2×10^5^ BHK21 cells were transfected with the three kinds of replicon plasmids (0.4 µg each), respectively, and pRL-TK plasmid (0.1 µg) as an internal control. The luciferase activity assays were performed 24 h post transfection. Error bars represent standard deviations of the mean of three experiments.

Although a large number of protein-protein interactions were detected for SARS-CoV in virtue of the large-scale screening analysis in mammalian two-hybrid system, the roles of these interactions in the viral replication and transcription were still not clarified. To obtain more clues to the general roles of individual proteins, we constructed a SARS-CoV replicon (Rep-SCV-luc/neo) that expresses the firefly luciferase gene, a sensitive reporter, under the control of M gene transcription regulatory sequence (TRS) as described in the Materials and Methods section (Fig. 3A). The effects of individual proteins or protein domains provided in *trans* on the replication/transcription of the SARS-CoV replicon were evaluated. Any effect of the protein provided in *trans* on the luciferase expression levels could in principle be due either to changes in the extent of the replication, the transcription, or a combination of both.

To evaluate the reporter gene expression of the replicon generated in this work, the BAC plasmid encoding Rep-neo/luc (pBAC-Rep-SCV-neo/luc) was transfected into BHK21 cells. A significant increase in luciferase activity, in comparison with that of the parental replicon plasmid pBAC-SARS-CoV-REP was observed (Fig. 3B). As the luciferase gene is located in the 3′-end of the genome and under the control of a viral TRS, it can only be expressed from subgenomic RNAs but not from the viral genome RNA. Therefore, expression of luciferase was expected to reflect the genome replication and transcription of SARS-CoV replicon. To further clarify whether the luciferase expression of Rep-SCV-luc/neo derived from the viral replication and transcription processes but not from nuclear splicing products of the large viral replicon RNA during the CMV promoter-driven transcription in nucleus, the positive and negative subgenomic RNAs were examined according to the strategy described in our previous work [39] and the existence of correct subgenomic RNAs was confirmed (data no shown). Moreover, when the gene segment between two MluI restriction sites in viral genome, which encodes nsp4–nsp11 and part of nsp12, was deleted, the construct pBAC-Rep-SCV-ΔMlu-luc/neo produced much lower levels of luciferase expression although it still retained detectable level of luciferase activity (Fig. 3B). To analyze the luciferase expression from the MluI deletion mutant, the identity of the luciferase mRNA was sequenced. The results showed that a low level of luciferase mRNA was generated either by cryptic promoters or by splicing of cryptic introns within the replicon transcripts in nucleus but not in the mRNAs derived from the cytoplasmic replication and transcription of the replicon (data not shown). Taken together, these results showed that the reporter-containing SARS-CoV replicon could be used as a model system for analyzing the functions of viral proteins.

**Figure 4:**
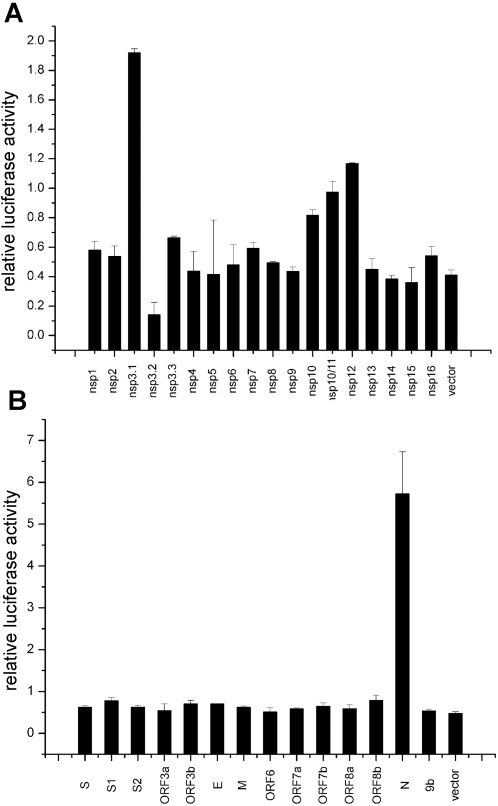
Impacts of viral proteins provided in *trans* on the replication/ transcription of SARS-CoV replicon. (A) Nonstructural proteins; (B) Structural and accessory proteins. 2×10^5^ BHK21 cells were transfected with pBAC- Rep-SCV-luc/neo (0.25 µg), the viral protein expression plasmids (0.2 µg each) and pRL-TK (0.05 µg) as an internal control. The corresponding empty vectors (0.2 µg each) were used as the negative controls. The luciferase activities were measured 24 h post transfection. Error bars represent standard deviations of the mean of three experiments.

To study the effect of SARS-CoV proteins on replication and transcription, individual viral proteins were co-expressed in *trans* with the SARS-CoV replicon. Renilla luciferase was employed as an internal control reporter to normalize the transfections among different wells (Fig. 4). Though most of the viral proteins did not exert significant impact on the replication and transcription of the replicon, the nsp3.1 containing X and SUD domains, and the RNA polymerase nsp12 (Fig. 4A), and nucleocapsid N (Fig. 4B) significantly increased the luciferase activity, whereas nsp3.2 containing the papain-like (PL) proteinase domain reduced the viral replication and transcription. Other proteins, such as nsp10/11, also showed a minor but significant increased expression. These results confirmed that the function of N gene fragment was not resulted from protein 9b that is internally nested in N sequence, as 9b showed no obvious impact on the replication and transcription of replicon when it was co-expressed (Fig. 4B). In addition, these results indicate that nucleocapsid protein, nsp3 and RNA polymerase may possess a dominant function in *trans*, while the PL proteinase is involved in important *cis* functions like cis-processing of the large viral polyprotein.

### Trans-activation activity of N protein at the early stage of genome replication or transcription of SARS-CoV

**Figure 5:**
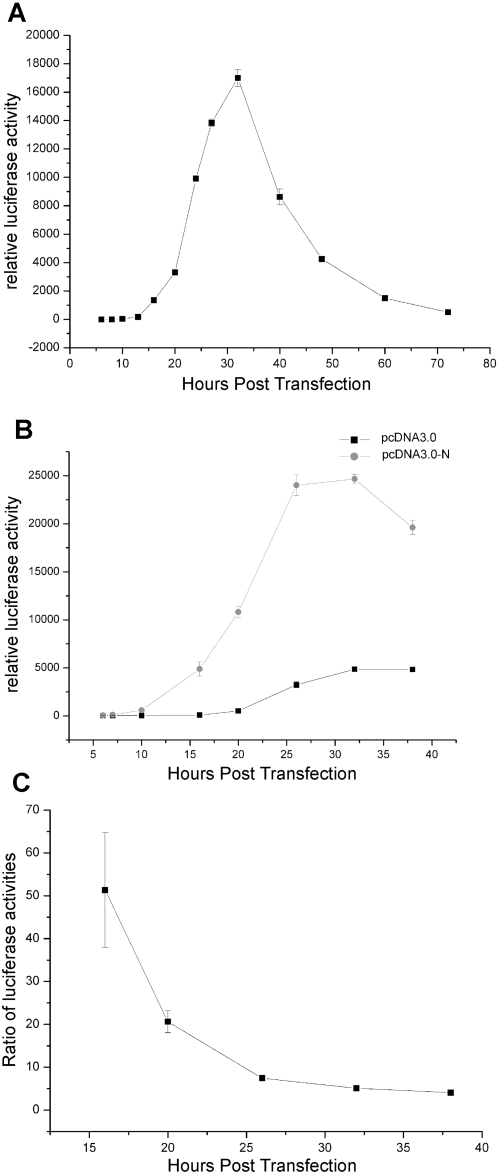
The effect of additional N protein on viral genome replication and transcription. (A) Kinetics of luciferase activity of the reporter replicon. 2×10^5^ BHK21 cells were transfected with pRL-TK plasmid (0.1 µg) and pBAC-Rep-SCV-luc/neo (0.4 µg). After transfection, the cells were collected for luciferase assays at different time points (6 h to 72 h). (B) Reporter gene activity in presence or absence of additional N protein provided in *trans*. 2×10^5^ BHK21 cells were transfected with pRL-TK plasmid (0.05 µg), pBAC-Rep-SCV-luc/neo (0.25 µg) and pcDNA3.0-N (0.2 µg) or pcDNA3.0 (0.2 µg). After transfection, the cells were harvested for luciferase assays at different time points (6 h to 38 h). (C) The ratios of luciferase activities of Rep-SCV-luc/neo in presence of N protein related to that in absence of N protein at different time points (6 h to 38 h). Error bars represent standard deviations of the mean of three experiments.

To make further analysis on the enhancement effect of N protein on the viral replication and transcription, we examined the dynamic changes of the luciferase activity expressed from the reporter replicon Rep-SCV-luc/neo. When the replicon plasmid pBAC-Rep-SCV-luc/neo was transfected alone, the luciferase activity could be detected 13 h post transfection (more than 10 times compared with background levels) while the highest value of activity was reached around 32 h post transfection (Fig. 5A). The luciferase activity became stable 72 h post transfection and the activity level was as low as that of 14 h post transfection (Fig. 5A).

To study the role of N protein provided in *trans*, we next transfected cells with equal amount of the reporter replicon together with either N protein expression construct (pcDNA3.0-N) or the empty vector pcDNA3.0. Luciferase activity became detectable at 7 h post transfection when N protein was provided in *trans* (Fig. 5B), which was 9 h earlier than similar transfection without additional N protein (Fig. 5B). It was also observed that the luciferase activity was significantly enhanced in the first 40 h post transfection (Fig. 5B). The ratios of luciferase activities with and without co-expression of N protein at different time points post transfection were shown in Fig. 5C. The ratios became lower step by step from over 50 times at 16 h post transfection to around 4 times at 38 h post transfection (Fig. 5C). The corresponding ratios for nsp3.2, nsp3.1 and nsp12 were also investigated and no similar phenomena were observed (data not shown). Collectively, these data showed that the nucleocapsid N protein provided in *trans* could enhance the efficiency of SARS-CoV genome replication and transcription at early stages.

## Discussion

Coronaviruses have the largest RNA genome known, which encodes a large number of proteins that are involved in viral replication, assembly, and other important functions that are essential to viral amplification in host cells. Except for some viral proteins that might perform their activities individually, most of the viral proteins could associate with other proteins or themselves to carry out their functions, indicating that the interactions between these proteins may play a crucial role during the viral life cycle. For SARS-CoV, at least 17 proteins, including 16 non-structural proteins and the structural nucleocapsid protein, are most likely involved in the replication process [12], [53].

In this study, a mammalian two-hybrid system was used to determine the genome-wide matrix-based protein-protein interactions of SARS-CoV. In total, 40 different interactions for 28 predicted mature proteins were observed, and most of them have not been previously reported. Interestingly, 32 percent of the interactions tested could be confirmed by pull down assays. To our knowledge, this is the first genome-wide protein-protein analyses for an intact viral ORFeome by using mammalian two-hybrid system. In our screen, 1.4 protein interactions per viral protein on the average was detected, and this result is in the upper range of the detection rates of viral protein interactions obtained by yeast two-hybrid systems [58]. Therefore, this study indicates that mammalian two-hybrid system can also serve as a convenient system to detect viral protein interactions of the whole ORFeomes. In addition, mammalian two-hybrid system may have some advantages over yeast two-hybrid system for detecting genuine viral protein interactions due to the native posttranslational modifications and folding of the proteins.

Very recently, two large-scale analyses of protein interactions of SARS-CoV were carried out by using yeast two-hybrid systems [9], [10]. In the report by von Brunn et al, 70 pairs of interactions were revealed, among which 30% could be verified by co-immunoprecipitation [9]. In the work by Imbert et al, 17 pairs of interactions were observed for non-structural proteins, about half of which were related to nsp3 [10]. Surprisingly, none of the interactions revealed in the two studies was overlapping although they both employed the yeast two-hybrid system for the screening. In contrast, overlapping interactions could be identified between that detected by mammalian two-hybrid system in current work and that by different yeast two-hybrid systems. There are 9 pairs of interactions found in our work that are overlapping with that of von Brunn et al, and they are nsp5 with nsp5, nsp7 and nsp8, nsp7 with nsp7, nsp8 and nsp9, nsp8 with nsp8, nsp9 and 9b. There are 4 pairs of interactions found in this study that are identical to that of Imbert et al. and they are nsp10 with nsp14 and nsp16 in both directions. Interestingly, all the overlapping interactions represented strong interactions in the mammalian two-hybrid assays and could be verified by biochemical assays.

In the two previous studies with yeast two-hybrid system, four proteins (nsp2, nsp3, nsp8 and 9b) were shown to have a wide range of interactions with other viral proteins, but these phenomena could not be observed in mammalian two-hybrid assays. In contrast, a number of interactions including N-N and nsp15–nsp15 were detected in current study and proved previously by other studies [54]–[57] but they could not be revealed by the yeast two-hybrid systems [9], [10]. Intriguingly, the two strong interactions nsp10–nsp14 and nsp10–nsp16 identified in mammalian two-hybrid system was also revealed in a yeast two-hybrid system by Imbert et al. [10] but not by von Brunn et al [9]. Although it is difficult to judge which system offers more biologically relevant results, great caution should be taken when interpreting the interaction data as both two-hybrid screenings as well as biochemical methods may generate false positive and false negative interactions. Nevertheless, the above comparative analysis of overlapping interactions of different systems may suggest that mammalian two-hybrid system could provide more reliable assays for detecting human viral protein interactions. In other scenario, the profiles of SARS-CoV protein interactions revealed by mammalian two-hybrid system (in this work) and yeast systems [9], [10], respectively, may be complementary to each other and jointly serve as a framework for further characterization of protein-protein interactions and their biological functions on coronavirus replication cycle.

Similar to that of yeast two-hybrid system, the detectable protein interactions all take place in the nucleus whereas, on the contrary, all positive-stranded RNA viruses replicate in cytoplasm, the natural location for viral proteins. Thus, the two-hybrid systems may have obvious limitation on detecting proteins that contain transmembrane domains or change to abnormal conformations in acidic conditions, and this may represent one reason for generating false negative and false positive interactions. In current screen, only few interactions were detected for the SARS-CoV membrane proteins such as nsp4, M, E and 3a proteins, and no interactions could be detected for nsp1, nsp6, spike and the small transmembrane protein 6, possibly reflecting the limitation of the two-hybrid system. Therefore, using shorter or random cDNA fragments by removing the transmembrane and other inhibitory domains may help detect more interactions in this system.

In this study, we have detected several interactions between accessory proteins and replicase proteins, for example, 3b with nsp8, nsp12, nsp13 or nsp14, 7b with nsp9 or nsp16, 8b with nsp9, and 9b with nsp8. Interestingly, among these interactions those between 3b with nsp8 and nsp13 were confirmed by pull-down assays. However, previous studies showed that deletions of ORFs 3b, 7b, 8b and 9b alone or in combination with other virus-specific proteins did not significantly influence the level of viral RNA and replication efficiency in cell culture and mouse models [48], [49], suggesting either that these interactions may not play significant roles in viral replication or that these systems are not sensitive enough to detect minor differences. In any case, it would be reasonable to speculate that such interactions may contribute to the virus-host interplay and hence to the viral pathogenicity.

The interactions between nsp10 with nsp14 and nsp16 showed bi-directionality in the mammalian two-hybrid analyses, which could be confirmed in pull-down assays and were also revealed in one of the two yeast-hybrid analyses [10]. Recently, nsp10 was shown to form a dodecameric RNA-binding protein complex [26], [27] involved in regulation of RNA synthesis and polyprotein processing [25], [59]. Nsp14 and nsp16 were proved to play an essential role in viral replication and transcription as shown by mutational analysis [37]. Considering that nsp14 has exoribonuclease activity and could play an essential role in RNA proof-reading activity [30]–[32], and that nsp16 is involved in the viral methyltransferase activity and RNA capping [34], [36], the interactions of nsp10 with nsp14 and nsp16 may indicate that nsp10 could play an important role in the formation of replication complex bound to RNA (i.e., nsp10 could mediate the binding of nsp14 and nsp16 to the RNA) and in the replication/transcription processes. Although no interactions were detected for nsp11, which was predicted to be small polypeptide with 13 amino acids, fusion of nsp11 with nsp10 strengthened the respective interactions of nsp10 with nsp14 and nsp16. These observations may imply that nsp11 could act as a cofactor for nsp10 and the predicted cleavage site between nsp10 and nsp11 may be inefficient in vivo, thus resulting in the production of nsp10/nsp11 fusion protein in cells. Indeed, no evidence for existence of the fully processed nsp11 has been presented in published researches. However, such speculation needs to be confirmed by further investigations.

Several interactions detected in current work have also been observed in previous studies. For example, nsp7 and nsp8 could associate with each other and thus form a hexadecameric supercomplex with a central channel that has dimensions and positive electrostatic properties favorable for nucleic acid binding [21]. Consistent with our results, self-interactions of N protein and nsp15 have also been revealed and the results indicate that these proteins have the propensity to oligomerize [56], [57], [61]. In this study, a SARS-CoV replicon containing a sensitive luciferase reporter was constructed, with its expression under the control of M gene TRS. In this situation, the reporter activity would indicate the efficiency of both genome replication and transcription. When individual viral proteins were provided in *trans* to the reporter replicon, most proteins did not exert significant influence on the reporter activity, with exception of nsp3, nsp12 (polymerase), and nucleocapsid protein N. As protein nsp3 is a large multidomain protein, different domains were tested separately in this system. While nsp3.1 that covers domains predicted for adenosine diphosphate-ribose 1″-phosphatase [12] and methyltransferase [35] could enhance the reporter activity, the nsp3.2 had a negative effect, indicating the domains in nsp3.2 may play structural roles in the formation of replication/transcription complex and could behave in a dominant-negative manner when separated from other domains. In accordance with this hypothesis, nsp3.2 was found to associate with itself, nsp4 and nsp12 in this work.

N protein of coronaviruses increases the rescue efficiency of coronaviruses from infectious RNA transcripts [37], [49], [62] and is required for efficient genome replication [53], [63]. In this study, we showed that N protein was not absolutely essential for starting the replication but may play important roles at the early stages of genome replication and/or transcription because the reporter replicon in absence of additional N protein provided in *trans* also resulted in obvious replication and transcription but in lower efficiency and delayed manner. The enhancement effects of N protein phased out at late stage probably when the N protein expressed from the genome had accumulated to certain level. Although the exact mechanisms for N-mediated stimulation activity at early stages are not known, several scenarios could be envisaged, such as protecting viral genome RNA, increasing the translation efficiency of viral RNA, stabilizing replication/transcription protein complex and inhibition of cellular innate immune responses, as is supported by the recent reports on diversified functions of N protein [64], [65].

In summary, current work constructed an interaction network of SARS-CoV proteins and established a sensitive replicon for studying the functions of individual viral proteins. The intraviral protein interactions identified in this study, in combination with the data obtained by using other systems, could serve as a basis for further studies of viral protein functions and molecular mechanisms of the genome replication/transcription processes of coronaviruses.

## Materials and Methods

### Cells and viral cDNAs

African green monkey kidney (Vero E6) cells, baby hamster kidney (BHK21) cells and 293T cells were grown and maintained in Dulbecco's modified Eagle medium and modified Eagle medium (Gibco Invitrogen), respectively, supplemented with 10% heat-inactivated fetal bovine serum and 100 U/ml of penicillin and 100 µg/ml of streptomycin (Gibco Invitrogen). Viral cDNAs encoding individual SARS-CoV ORFs were generated and described in our previous work [39], [66].

### Cloning and expression of the SARS-CoV ORFs

The primers for individual ORFs were designed according to the genome sequence of SARS coronavirus strain WHU (GenBank accession number: AY394850) with restriction sites which were compatible for the downstream subclonings. The reagents for PCR were 0.2 µM dNTPs, 0.4 µM forward and reverse primers, ∼10 ng template DNA and 1 U of KOD DNA polymerase (TOYOBO) in 50 µl reaction system. The amplification conditions were 94°C for 2 min and 30 cycles of 94°C for 15 sec, 52°C for 15 sec and 68°C for 3 min, followed by 68°C for 10 min and 4°C for 10 min.

After the separation by agarose gel electrophoresis, PCR fragments were recovered from the gels by DNA extraction kit (OMEGA BIO-TECH) and cloned into pGEM-T vector (Promega) after A-tailing reaction according to standard protocols. The sequences of positive clones examined by restriction and PCR analyses were confirmed by DNA sequencing on ABI 3730 DNA Analyzer (Applied Biosystems) and CEQ™ 8000 Sequencer (Beckman Coulter). Subsequently, the coding sequences were cut from pGEM-T constructs with appropriate restriction enzymes and cloned into the mammalian two-hybrid vectors pM and pVP16 (Clontech), *Escherichia coli* protein expression vectors pET30a (Novagen), pGEX-6P-1 (Amersham Biosciences) and pMAL-c2X (New England Biolabs), and mammalian expression vectors pcDNA3.0 (Invitrogen Corporation), pCMV-Tag2b (Stratagene Corporation) and pCIH that was generated by adding an ATG-HA-tag oligo at the XhoI-EcoRI sites of vector pCI-Neo (Promega). Details of the cloning processes and various constructs can be provided upon request.

### Mammalian two-hybrid analysis

The reporter construct pG5-luc was reconstructed from pG5CAT (Promega) by replacing chloramphenicol acetyl transferase (CAT) gene with luciferase gene sequence. For protein interaction analysis, 0.3 µg of DNA-binding domain fusion constructs in plasmid pM, 0.3 µg of transcriptional activation domain fusion constructs in plasmid pVP16, 0.15 µg of reporter construct pG5-luc and 0.1 µg of vector pRL-TK (Promega) as internal control were transfected into 293T cells by calcium phosphate transfection method. One day before transfection, 293T cells were seeded in 24-well plate with ∼1×10^5^ cells per well. On the following day, the DNA of specified amount and 2.5 µl of 2.5 M CaCl_2_ were diluted with deionized H_2_O to reach a final volume of 25 µl. After an incubation for 5 minutes, 25 µl of 2×HBS solution (50 mM HEPES, 10 mM KCl, 12 mM Dextrose, 280 mM NaCl, 1.5 mM Na_2_HPO_4_, pH 7.05) was added and mixed. The calcium phosphate-DNA solution was added dropwise to the cell culture medium while swirling the plate gently. After incubation for 6 h at 37°C with 5% CO_2_, the medium was replaced with fresh one. 24 h later, the luciferase assays were performed by Dual-Luciferase reporter Assay System (Promega Corporation) according to the provider's instructions. Several control constructs were adopted in the analysis, including pM-53 which encodes the p53 protein, pVP16-T which encodes the SV40 large T antigen, and the empty vectors pM and pVP16. The known interaction between p53 and T antigen was used as positive control, and the combinations of test construct and one control plasmid were assigned as negative controls. The assays on each pair of plasmid combinations were repeated at least 4 times and 3 wells were examined per assay.

### GST-pull down assays

Recombinant glutathione S-transferase (GST)-fusion proteins and maltose-binding protein (MBP)-fusion proteins were expressed in *E. coli* BL21 and DH5α, respectively. Bacterial cells harvested were resuspended in phosphate-buffered saline (PBS, 10 mM sodium phosphate, 150 mM NaCl, pH 7.5) and lysed by sonication in ice. The lysate was subsequently clarified by centrifugation at 12,000 g for 30 min at 4°C. GST fusion proteins and GST protein immobilized on glutathione-Sepharose were coated firstly with MBP protein and were subsequently incubated with the lysate for MBP-fusion proteins in PBS containing protease inhibitor cocktail. After incubation with rotation for 2 h, the resin of glutathione-Sepharose was precipitated by brief spin and washed by PBS at least for 5 times. The resin was re-suspended in SDS-PAGE loading buffer and heated at 100°C for 5 min. The samples were separated in 10% SDS-PAGE and transferred to PVDF membrane following the standard procedures of Western-blotting. Anti-MBP rabbit serum (New England Biolabs, dilution 1∶5000) was used in the immunoblot analysis to identify MBP-fusion proteins.

### Construction of a replication and transcription report system for SARS-CoV

The reporter system was based on the primary SARS-CoV replicon (pBAC-SARS-CoV-REP) [37]. To construct a reporter-containing replicon including a selection gene for mammalian cells, the luciferase and neomycin fusion gene cassette was amplified by PCR from a hepatitis C virus replicon provided by Dr. Ralf Bartenschlager [67]. The luciferase-neomycin gene sequence was fused with and thus controlled by the transcription regulation sequence (TRS) of SARS-CoV M gene. The fused sequence containing TRS/M-luciferase-neomycin cassette was inserted between AscI and BamHI sites in pBAC-SARS-CoV-REP, resulting in the reporter replicon construct pBAC-Rep-SCV-luc/neo. The cloning details can be provided upon request. Several transfection strategies and reagents were examined for the transfection of pBAC-Rep-SCV-luc/neo and the relatively higher transfection efficiency was obtained with FuGENE HD Transfection Reagent (Roche Applied Science). Several cell lines were also examined and BHK21 turned out to better support the replication/transcription of Rep-SCV-luc/neo and produced consistent results.
